# Comparing the medication costs of treating patients with schizophrenia who use cannabis with those who do not

**DOI:** 10.4102/sajpsychiatry.v30i0.2211

**Published:** 2024-04-15

**Authors:** Nikhil Nowbath, Nada Abdelatif, Gian Lippi

**Affiliations:** 1 Department of Psychiatry, School of Medicine, Faculty of Health Sciences, University of Pretoria, Pretoria, South Africa; 2 Department of Biostatistics Research Unit, South African Medical Research Council, Pretoria, South Africa

**Keywords:** medications costs, schizophrenia, cannabis, adult males, inpatient treatment

## Abstract

**Background:**

Cannabis use is more prevalent among people with schizophrenia than in the general population. This usage detrimentally impacts disease prognosis, contributing to escalated admissions, heightened severity of psychotic symptoms, and reduced medication response. The recent decriminalisation of cannabis in South Africa may lead to an upsurge in usage, consequently intensifying the strain on mental healthcare services.

**Aim:**

This study aimed to compare the medication costs of patients with schizophrenia depending on cannabis use.

**Setting:**

Weskoppies Hospital, Pretoria, South Africa, 2018–2019.

**Methods:**

Data pertaining to medication expenses during the 2018–2019 period were acquired from the hospital pharmacy. Data were collected from 114 patient records to form two equal cohorts: one exposed to cannabis and the other non-exposed, as indicated by urine drug screens or admission of cannabis use. Medications prescribed from admission to time of being ready for discharge were recorded and corresponding costs were calculated.

**Results:**

Patients who were exposed to cannabis had higher medication costs (R 516.47) than patients who were non-exposed (R 328.69) (*p* = 0.0519), over the whole admission period.

**Conclusion:**

Cannabis exposure escalates the financial burden of treating schizophrenia at Weskoppies Hospital. This might be attributed to failure of cost-effective, first-line medications prompting the prescription of costlier, second-line alternatives or higher prescribed dosages.

**Contribution:**

This study contributes to findings that it is more expensive to treat patients with schizophrenia who have relapsed, if they are using cannabis. This finding has future cost implications when budgeting for pharmacotherapeutic treatment.

## Introduction

Schizophrenia is a chronic, debilitating mental illness affecting an estimated 20 million people worldwide.^1^ Schizophrenia is a leading cause of disability, resulting in tremendous health, social and economic burdens for patients, caregivers and the wider society. Psychotic symptoms and disorders, including schizophrenia, have been linked to cannabis use.^2^ The nature of the association between cannabis and schizophrenia is complex and has many confounding factors. There is some debate as to whether cannabis can cause schizophrenia, or whether the association between the two represents a shared genetic vulnerability to both cannabis use disorder and schizophrenia.^2^ Cannabis use during first episode psychosis is associated with a substantially higher rate of acute psychiatric admissions and may be associated with increased healthcare costs.^3^

Globally, cannabis is the most commonly used illicit drug. A 2017 survey of 39 207 participants from South Africa found that 1 in 10 participants had used cannabis in the preceding 3 months.^4^ A study in KwaZulu-Natal at a tertiary psychiatric hospital found that out of 370 people admitted with schizophrenia spectrum and other psychotic disorders, 48.9% (*N* = 180) reported current cannabis use and 51.1% (*N* = 188) reported lifetime cannabis use.^5^ Up to 60% of people with schizophrenia may use substances, with cannabis being the most widely used substance in this population (27% – 42%).^1^ This is considerably higher than cannabis use in the general population, which is estimated to be around 4%.^6^

The endocannabinoid system (ECS) includes receptors, ligands and enzymes. It regulates a number of physiological functions and mediates communication between different neurotransmitter systems. The ECS is widely distributed, and one of its two receptors, the cannabinoid type 1 (CB1)-receptor, is the most abundant G-protein coupled receptor in the brain. As a result of its wide distribution, modulation of the ECS has been found to impact emotional regulation, motivational behaviour and cognitive functioning.^7^

The endogenous cannabinoids are anandamide and 2-arachyldonoylglycerol (2-AG). They bind to the CB1 and CB2 cannabinoid receptors. The ECS is disturbed in schizophrenia, specifically with a polymorphism of the CB1 receptor gene.^8^ Cannabinoid type 1 receptors are also found in higher densities in the prefrontal cortex, hippocampus and basal ganglia in people with schizophrenia.^8^

Tetrahydrocannabinol (THC) is an exogenous cannabinoid and is the main psychoactive ingredient found in cannabis. It binds to both the CB1 and CB2 receptors. Anandamide has been found to have protective properties in people with schizophrenia. Higher cerebrospinal fluid (CSF) levels of anandamide have been associated with fewer and less severe psychotic symptoms.^9^ Cannabis use has been found to decrease the levels of anandamide present in the CSF through the actions of THC; thus, people with schizophrenia who use cannabis are more likely to have more severe psychotic symptoms.^9^

Studies have also shown that cannabis use may aggravate psychosis in patients with schizophrenia.^3^ Among patients with schizophrenia, cannabis use has been associated with reduced adherence to treatment, higher relapse rates leading to readmission, and increased severity of psychotic symptoms.^10^ People living with schizophrenia who use cannabis also have a worse prognosis.^11^ Patients with schizophrenia are often admitted to psychiatric hospitals as involuntary users because of lack of insight and capacity. They pose a higher risk to themselves and others in society. The total cost of schizophrenia to society includes direct medical costs and indirect costs. Indirect costs may be incurred by caregivers as they may need to pay for care or even give up their jobs to care for patients with schizophrenia.^12^

Direct medical costs include treatment costs of which 25% represent medication costs for treating schizophrenia.^13^ Antipsychotics are the most commonly prescribed medications for treating schizophrenia.^13^ Aside from exacerbating psychotic symptoms, cannabis use may reduce the efficacy of antipsychotic medications used to treat schizophrenia. Patients with schizophrenia who use cannabis are thus more likely to require higher doses of antipsychotics and suffer from more side effects.^14^

Policymakers often consider economic data when setting priorities and making spending choices in resource-limited settings. Currently, there are no reviews of costs related to mental disorders in South Africa and few reviews that examine costing aspects at a global level.^14^ Here, we compare the medication costs between schizophrenia patients who use cannabis and those who do not use cannabis. The decriminalisation and possible legalisation of cannabis on top of an already strained healthcare system may further deplete scarce resources. The findings may aid legislators when deciding on the possible legalisation of cannabis.

## Research methods and design

### Study design

This was a quantitative, retrospective case control study. Data were collected from patients diagnosed with schizophrenia admitted to Weskoppies Hospital between 01 January 2018 and 31 December 2019. The patients were divided into two groups: those who had used cannabis shortly before or during admission and those who had not been exposed to cannabis shortly before or during admission. Cannabis use was screened using the Instant View, Multidrug Screen Urine Test, which has a sensitivity of 82.1% and a specificity of 87.9%.^15^

Data collected from patient files included age, relevant comorbidities, duration of admission, and whether it was their first admission or not. For each patient, we recorded medication prescribed from admission until patients were discharge ready. Prescription information included antipsychotic medication, medication used to treat side effects of antipsychotics, and medication used for acute sedation. The price of these medications was obtained from the pharmacy.

### Study population

Data were collected for adult males (>18 years old), with schizophrenia, who were admitted to and discharged from Weskoppies Hospital during the study period. There were 114 participants, 57 of whom were exposed to cannabis and 57 who were not exposed to cannabis. Only patients admitted to acute adult psychiatric wards of the hospital were included in the study. Patients who tested positive for illicit substances other than cannabis on the Instant View, Multi Drug Urine Test were excluded.

### Data collection

Files that met the inclusion criteria were obtained from the Weskoppies Hospital archives. A specially designed data capturing sheet was used to record admission duration, number of previous admissions, first admission or readmission, and relevant comorbidities. The average daily dose of medication for the duration of admission and the total cost thereof were calculated using information from the pharmacy. Medication costs of monthly prescriptions, such as depot antipsychotic medications, and sedatives that were prescribed on an as needed basis, were calculated by multiplying the cost of the medication by the total number of doses received. A separate data capturing sheet was designed to capture the price of medication paid by the hospital for the period 2018–2019.

### Data analysis

Data were analysed using Stata version 16.1 (StataCorp. 2019. Stata Statistical Software: Release 16. College Station, TX: StataCorp LLC). Descriptive statistics were presented using frequency tables and percentages, as well as summary statistics. This included means (along with the standard deviations) and medians (along with ranges and interquartile ranges) that were calculated for continuous variables that were normally distributed. Medication costs for the duration of admission of antipsychotics and sedatives, as well as the duration of treatment, were compared between cannabis exposed patients versus patients who did not use cannabis. The Student’s *t*-test was used to compare the two groups. Bootstrapping was carried out to test the robustness of the Student’s *t*-test because cost estimates were non-normally distributed. For treatment duration, quantile regression was used to determine whether the median of the two groups of patients differed significantly. A *p* < 0.05 was considered significant.

### Ethical considerations

Ethical clearance to conduct this study was obtained from the University of Pretoria Faculty of Health Sciences Research Ethics Committee (No. 102/2022).

## Results

Table 1 shows the cost of oral medications at the hospital for the period 2018–2019. Using this information, the daily cost of a specific dose of medication was calculated. Table 2 has similar information regarding the cost of injectable medications.

**TABLE 1:** The cost of oral medications for the period 2018–2019.

Medication	Dose (mg)	Pills	Cost per box	Cost per pill
Amisulpride	50	30	55.44	1.85
Amisulpride	200	30	153.78	5.13
Chlorpromazine	25	28	17.3	0.62
Chlorpromazine	100	56	82.85	1.48
Clozapine	25	100	38.33	0.38
Clozapine	100	100	122.98	1.23
Haloperidol	1.5	60	17.63	0.29
Haloperidol	5	50	24.39	0.49
Olanzapine	2.5	28	14.49	0.52
Olanzapine	5	28	26.2	0.94
Olanzapine	10	28	24.31	0.87
Risperidone	0.5	30	5.51	0.18
Risperidone	1	30	5.55	0.19
Risperidone	2	30	5.8	0.19
Risperidone	3	30	7.25	0.24
Quetiapine	25	100	31.33	0.31
Quetiapine	100	90	67.99	0.76
Quetiapine	200	60	85.05	1.42
Quetiapine	300	60	160.39	2.67
Alprazolam	0.5	30	16.88	0.56
Clonazepam	0.5	84	41.22	0.49
Clonazepam	2	84	95.92	1.14
Diazepam	5	100	10.09	0.10
Lorazepam	1	100	106.06	1.06
Lorazepam	2.5	100	166.44	1.66
Oxazepam	10	100	45.14	0.45
Oxazepam	30	100	61.23	0.61
Orphenadrine	50	56	39.25	0.70
Propranolol	10	250	28.6	0.11

**TABLE 2:** The cost of injectable medications for the period 2018–2019.

Medication	Strength (mg)	Price per ampule
Flupenthixol decanoate	20	37.42
Paliperidone extended release	50	1019.64
Paliperidone extended release	75	1513.03
Paliperidone extended release	100	2299.83
Paliperidone extended release	150	2299.83
Zuclopenthixol acetate	50	98.41
Zuclopenthixol decanoate	200	52.12
Risperidone extended release	25	571.76
Risperidone extended release	37.5	848.43
Risperidone extended release	50	1131
Diazepam	5	3.87
Lorazepam	4	76.76
Biperiden	5	47.43

Table 3 shows the number of participants in each group who were treated with a specific medication. The most prescribed medication was risperidone, which was prescribed to 59 patients (cannabis unexposed: *n* = 28, cannabis exposed: *n* = 31). Haloperidol was the other first-line oral antipsychotic and was prescribed to 13 and 8 patients in the cannabis unexposed and exposed groups, respectively. Olanzapine was generally the next antipsychotic prescribed when a patient failed on first-line options and was prescribed to 7 and 15 patients in the cannabis unexposed and exposed groups, respectively.

**TABLE 3:** Number of participants in each group who were treated using a specific antipsychotic.

Medication	Cannabis unexposed	Cannabis exposed	Total
**Oral antipsychotics**			
Aripiprazole	1	0	1
Quetiapine	1	0	1
Haloperidol	13	8	21
Risperidone	28	31	59
Olanzapine	7	15	22
Amisulpride	1	1	2
Clozapine	3	4	7
**Injectable antipsychotics**			
Zuclopenthixol decanoate	18	21	39
Flupentixol decanoate	8	25	33
Paliperidone	0	2	2
Risperidone	1	0	1

Clozapine is most commonly prescribed for patients with treatment-resistant schizophrenia and was prescribed for three patients who had not used cannabis, compared with four patients who had used cannabis.

Long-acting injectable antipsychotics were prescribed to 75 patients in total; 27 and 48 patients in the cannabis unexposed and exposed groups, respectively.

For the 114 participants, the mean cost of medication during admission was R 422.58 (Table 4). Medication costs for the cannabis exposed and unexposed groups were R 516.46 and R 328.69, respectively (*p* = 0.0519). Patients in the cannabis exposed groups had longer admission durations than patients not exposed to cannabis (Table 4). The mean age of patients was 35.48 years. Patients in the cannabis exposed group were younger (31.68 years) than patients in the cannabis unexposed group (39.28 years). There were 20 (17.54%) index presentations: 12 (21.05%) in the cannabis exposed group and 8 (14.04%) in the cannabis unexposed group.

**TABLE 4:** Cost of treatment and duration of admission for schizophrenia patients at Weskoppies Hospital, 01 January 2018–31 December 2019.

Group	Cost of treatment (ZAR)	Admission duration (days)
Both groups	422.58	54.88
Cannabis exposed	516.47	57.57
Cannabis unexposed	328.69	53.09

## Discussion

We compared the cost of treating patients with schizophrenia who used or did not use cannabis shortly before or during admission to Weskoppies Hospital. The mean medication costs during admission for patients who used cannabis were R 516.47, while patients who did not use cannabis had lower medication costs (R 328.69). For the total duration of admission, the difference in medication costs was R 187.78. When factoring in duration of admission, the daily cost of medication was R 9.13 per day for the cannabis exposed group and R 6.20 per day for the cannabis unexposed group, translating to a 47% increase in daily medication costs.

Previous studies have suggested that cannabis use increases the cost of managing schizophrenia by worsening the severity of psychosis and reducing the efficacy of prescribed medication.^9^ A poor response to first line treatment options leads to the prescription of costlier second options. In the study, patients were mostly prescribed risperidone, haloperidol and olanzapine. Risperidone and haloperidol were first line oral antiphyscotics and were largely prescribed to patients who did not use cannabis. In contrast, patients who used cannabis were largely prescribed risperidone and olanzapine. While the monthly costs of risperidone and haloperidol were close to R 14.40 per patient, the monthly cost of olanzapine was R 52.20 per patient, which is much pricier. In the study, if patients did not respond to any of these three oral antipsychotic options, they were prescribed a more expensive drug. In the study, four patients were prescribed either quetiapine, aripiprazole or amisulpiride, resulting in monthly costs ranging from R 145.50 to R 923.40 per patient. These figures demonstrate the difference in monthly costs between first line and second line medications. Clozapine was most likely prescribed to patients considered to be treatment-resistant. The monthly cost of clozapine prescribed at 400 mg per day is R 147.60.

Comorbid cannabis use disorder has been found to significantly increase the rate of medication non-adherence in patients with schizophrenia.^16^ The use of injectable antipsychotics has been found to improve medication adherence,^17^ which explains why injectable antipsychotics were prescribed more in the cannabis exposed group in our study.

Importantly, patients should be given adequate duration trials of first line medications and even slight improvements should be recorded before switching to second line options. A 2018 study reported that out of 100 patients who showed minimal improvement on the positive and negative syndrome scale (PANSS) after 2 weeks of treatment on risperidone, 86 showed significant improvement at 4 weeks of treatment.^18^ Even though risperidone is promising for patients with schizophrenia, cannabis use may reduce the efficacy of this first line antipsychotic and lead to the prescription of second line options.^19^ In 2017, THC was shown to reduce the available concentrations of risperidone and its active metabolite, 9-hydroxy risperidone, in the brain by increasing the expression of the ABC transporter P-glycoprotein (P-gp), which binds with risperidone and transports risperidone across the blood–brain barrier to the peripheral circulation.^19^ Interestingly, clozapine is not a substrate for P-gp and has been found to be a more effective antipsychotic for patients who have been exposed to cannabis.^10,19^ However, this study found that clozapine was only prescribed to four out of 57 patients who used cannabis. The low rate of clozapine use may be because of patients who responded adequately to first line treatment. There may also be a reluctance by prescribers to use clozapine because of its significant adverse effects and related need for frequent blood sampling, and a reluctance by patients to take oral antipsychotics because of poor insight into their illness.

Cannabis use is known to increase the severity of schizophrenia symptoms. In 1986, participants who were using cannabis during a 6-month observation period presented with significantly more intense delusional and hallucinatory activity than those who did not.^20^ Regular cannabis use has been found to cause neuroanatomical changes in the amygdala, hippocampus, prefrontal cortex and cerebellum.^21^ People who regularly use cannabis have higher concentrations of CB-1 receptors in these areas, and these areas are smaller than in non-cannabis users.^21^ These differences have also been observed among people with schizophrenia who use cannabis and those who do not.^22^

In this study, we noticed several anecdotal findings. Cannabis exposed patients had slightly longer duration of admission than patients in the cannabis unexposed group, although this duration was not significantly different. Currently, the evidence is inconclusive regarding the impact of cannabis use on admission duration, with some studies concluding that cannabis exposure is not a good predictor of length of stay,^23^ while other studies found that cannabis exposure was associated with increased duration of admission.^24^ In total, our study included 20 first-episode psychosis patients, of whom 12 (60%) were in the cannabis exposed group and 8 (40%) in the cannabis unexposed group. Current evidence suggests that up to 64% of first-episode psychosis patients have used cannabis.^25^ We also noticed that participants in the cannabis exposed group were roughly 7.5 years younger than patients in the cannabis unexposed group. Cannabis use has been associated with earlier onset of schizophrenia, as well as more frequent admissions,^26^ which might explain why participants in the cannabis exposed cohort were so much younger.

Despite the clear link between cannabis and psychosis, there has been a global shift towards the legalisation of cannabis. Unfortunately, the effects of cannabis on people who have schizophrenia are not always considered when debating the legalisation of cannabis.^27^ In 2018, the South African High Court ruled that using or possessing cannabis in a private place would not be a criminal offence, and South Africa became the first country in Africa to decriminalise cannabis.^28^ The decriminalisation and possible legalisation of cannabis may lead to more people with schizophrenia using cannabis, which will ultimately increase medication and subsequent treatment costs.

### Limitations

This study had a relatively small sample size of 114 patients. A larger sample size would reduce the confounding impact caused by grouping patients with first episode psychosis together with those experiencing multiple episodes of psychosis, as the difference in cost may be related to the stage of the illness as opposed to exposure to cannabis. There was no distinction made between patients on monotherapy and those on multiple agents. Polypharmacy could also be a confounder, leading to increased cost of treatment. The medication costs represented the prices for the period 2018–2019, and these figures are likely to change for each financial year. The cost per dose was calculated assuming that the most cost effective way of administering the dose to a patient was used. However, because of stock issues, there may have been occasions where a patient was given a dose according to what was available, for example, taking two risperidone 1 mg tablets, instead of one risperidone 2 mg tablet, which would confound the results.

This study was set at a single public institution in South Africa. The results cannot be generalised to other settings. The medication costs may be much higher at a private psychiatric hospitals or while consulting a private psychiatrist.

## Conclusion

The results show that cannabis exposure led to increased medication costs for people with schizophrenia from admission until patients were ready for discharge. The most likely contributing factor may be treatment failure of first line options brought on by cannabis use, which leads to the prescription of costlier second line options or higher dosages. The legalisation of cannabis may increase the burden on mental healthcare services, and further cost analyses should be performed. Patients with schizophrenia, as well as their caregivers, should be comprehensively educated on the potential impact of cannabis on the progression and treatment of their disease.
